# Application and Mechanisms of Internet-Based Cognitive Behavioral Therapy (iCBT) in Improving Psychological State in Cancer Patients

**DOI:** 10.7150/jca.82632

**Published:** 2023-07-03

**Authors:** Ping Bai

**Affiliations:** 1 Department of Operating Rooms, National Cancer Center/National Clinical Research Center for Cancer/Cancer Hospital, Chinese Academy of Medical Sciences and Peking Union Medical College, Beijing, 100021, China.; 2 Department of Operating Rooms, National Cancer Center/National Clinical Research Center for Cancer/Hebei Cancer Hospital, Chinese Academy of Medical Sciences, Langfang, 065001, China.

**Keywords:** Application, Mechanisms, Internet-Based Cognitive Behavioral Therapy (iCBT), Psychological, Cancer

## Abstract

This review article is an overview of the effectiveness of internet-based cognitive behavioral therapy (iCBT) in Improving Psychological State in Cancer Patients. iCBT's effectiveness has been investigated in treating and managing conditions like depression, psychiatric disorders, generalized anxiety disorder (GAD), panic disorder, obsessive compulsive disorder (OCD), post-traumatic stress disorder (PTSD), adjustment disorder, bipolar disorder, chronic pain, and phobias. iCBT's role in the treatment of medical conditions such as diabetes mellitus with comorbid psychiatric illnesses was also explored. We conducted a thorough literature search using PubMed, Embase, Google Scholar, and Wanfang with no restrictions on the date. iCBT's role in treating and controlling psychiatric illnesses in cancer patients has been established in the literature. Development and popularization of iCBT, treament forms of iCBT, platforms for iCBT, application of iCBT, strategies and efficacy of iCBT for insomnia in cancer patients, current status of iCBT application, and genetic researches on iCBT for anxiety disorders were all reviewed and discussed in this review. From the data compiled, we conclude that iCBT is useful in treating or improving psychological state in cancer patients.

## 1. Introduction

Oncological patients exhibit a substantial prevalence of concomitant psychiatric disorders and non-specific psychological distress^1^. The co-occurrence of psychiatric disorders and clinically significant psychological distress in this population may reach as high as 85%; however, it is postulated that less than 50% of afflicted cancer patients receive adequate identification and referral for appropriate care.

Psychiatric disorders arising in cancer patients may be correlated with elevated mortality rates. A national registry study examined over 1,600 esophageal cancer patients who underwent surgery without any previous psychiatric disorder history^2^. Following adjustments for potential confounding variables (e.g., age, marital status, tumor stage, and histology), the analysis revealed that all-cause mortality was higher among patients who developed psychiatric disorders, as opposed to those who remained psychiatrically unaffected (hazard ratio 1.7, 95% CI 1.2-2.3)^2^.

A minimum of one-third of cancer patients exhibit comorbid psychiatric disorders. A meta-analysis of eight observational studies encompassed 1,448 cancer patients assessed via validated instruments, determining that at least one psychiatric disorder (adjustment, anxiety, depressive, somatic symptom, or substance use disorders; or post-traumatic stress disorder) was present in 32%; significant heterogeneity was observed across the studies^3^. A subsequent cross-sectional study involving interviews of over 2,000 cancer patients ascertained that at least one mental disorder (e.g., adjustment, anxiety, depressive, somatic symptom, or substance use disorders) was present in 32%^4^ A national registry study comparing over 300,000 cancer patients and more than 3,000,000 individuals without cancer investigated the composite incidence of psychiatric disorders (adjustment, anxiety, depressive, somatic symptom, or substance use disorders) in both cohorts^5^. After controlling for age, sex, and education, the analysis indicated a higher incidence of psychiatric disorders among cancer patients compared to controls, with the most pronounced increase in mental disorders occurring during the first week following diagnosis, rendering cancer patients seven times more likely to develop a psychiatric disorder (hazard ratio 6.7, 95% CI 6.1-7.4)^5^.

Through substantial efficacy, cognitive behavioral therapy (CBT) frequently permeates depression treatment^6^. Investigating patients' beliefs, these approaches scrutinize cancer diagnosis and treatment perceptions, targeting maladaptive or counterproductive cognition that fosters helplessness and desolation. Therapeutic endeavors ensue, amending maladaptive thought schemas and instilling novel coping techniques (e.g., relaxation). Both collective and singular treatment modalities effectively alleviate depressive manifestations and distress, augmenting life quality. Cognitive-behavioral interventions facilitate patients in assuaging disproportionate fears, stimulating contemplation of diverse potential consequences. By directing patients towards controllable illness and treatment facets, and endorsing behavioral adjustments that preserve engagement and positivity, life quality may experience enhancement.

In the late 1990s, Internet-Based Cognitive Behavioral Therapy (iCBT) materialized as global researchers investigated digital psychotherapeutic methods^7^. Contextually scrutinizing iCBT's efficacy, conditions treated and managed include depression, generalized anxiety disorder (GAD), panic disorder, obsessive-compulsive disorder (OCD), post-traumatic stress disorder (PTSD), adjustment disorder, bipolar disorder, chronic pain, and phobias. Furthermore, iCBT's potential for managing medical afflictions like diabetes mellitus accompanied by psychiatric comorbidities has been probed^8^. This review article presents an exhaustive synthesis of iCBT's effectiveness in addressing psychiatric disorders and enhancing the psychological well-being of cancer patients. A comprehensive dissection will encompass iCBT's evolution and popularization, diverse treatment modalities, iCBT implementation platforms, applications, strategies and efficacy for insomnia in cancer patients, iCBT application's contemporary state, and genetic research concerning iCBT for anxiety disorders.

## 2. Elaboration on the Application of Cognitive Behavioral Therapy (CBT)

Cognitive Behavioral Therapy (CBT) constitutes a sophisticated psychotherapeutic approach, devised to rectify patients' erroneous cognitions, deleterious emotions, and adverse behaviors through the modification of their thought patterns, convictions, and actions^9^. The underpinning principle of CBT is that it is considerably challenging to directly alter patients' emotions; however, their feelings can be influenced by the manipulation of their thoughts and behaviors. CBT embodies an integrative therapeutic modality, combining the tenets of both cognitive and behavioral therapies. Professor Allen Baker pioneered CBT in the 1960s as a means to alleviate depressive symptoms, upon discerning the existence of “cognitive distortion traits” within the thought processes of individuals afflicted with depression^10^. Through meticulous clinical observations and empirical investigations, Baker formulated a cognitive theory of depression and validated the efficacy of CBT, premised on the notion that an individual's cognitions can exert a significant impact on their behaviors, and conversely^10^. Initially, CBT was predominantly employed in the treatment of patients suffering from mental disorders such as insomnia^11^, detrimental emotions, and pain^12^. In recent times, due to the escalating prevalence of cancer, CBT has been increasingly utilized in the management of cancer patients to enhance their overall quality of life by rectifying their inaccurate cognitions, emotions, and behaviors^13^. The implementation process of CBT encompasses the following stages:

### 2.1 Comprehension of Patients' Cognitions

The therapist engages patients in open-ended inquiries (e.g., “What brings you here? How do you feel at present?”) and encourages them to verbalize their innermost sentiments. This exchange of information enables the therapist to discern the patients' misconceptions and determine the most appropriate therapeutic intervention for their specific needs.

### 2.2 Cognitive Restructuring

The therapist addresses patients' misapprehensions pertaining to salient concerns, elucidates the potential ramifications of such negative ruminations on treatment outcomes, fosters patients' capacity for positive thinking and interaction with fellow patients, guides patients in examining alternative viewpoints through open-ended questions, and facilitates a critical appraisal of their behaviors and cognitions, thereby transforming the patients' emotional or cognitive processes.

### 2.3 Behavioral Intervention

The therapist directs patients to engage in thought exercises, document affirmative reflections, employ distraction techniques and progressive relaxation strategies, cultivate pertinent skills, and practice visualization, both during therapeutic sessions and in their daily lives. The therapist employs a comprehensive array of techniques to facilitate patients' engagement in adaptive behavioral practices, supporting them in modifying maladaptive patterns and fostering healthier coping strategies, both within the context of therapy sessions and in their daily lives. These techniques could be seen in **Table 1**.

## 3. Development and Popularization of iCBT

Cognitive Behavioral Therapy (CBT) constitutes a short-term psychotherapeutic approach targeting the mitigation of negative emotions and behaviors through the transformation of detrimental cognitions via thought and behavior modification. Internet-Based Cognitive Behavioral Therapy (iCBT), derived from Baker's cognitive model and the behavioral activation theory, endeavors to impart information and expertise akin to conventional CBT in a structured, standardized manner. iCBT's popularity stems from its acceptability, efficacy, and feasibility, with two primary modalities: therapist-guided iCBT and self-help iCBT. Therapist-guided iCBT typically comprises online coursework, assignments, and weekly 10-30 minute guidance sessions with a therapist through telephone or email correspondence^14^. Meta-analyses have demonstrated equivalent effectiveness between therapist-guided iCBT and traditional CBT for patients with depression across all age groups^14^. Self-help iCBT entails no therapist involvement, requiring participants to independently complete lessons and assignments according to a predetermined schedule. Presently, iCBT programs for depression treatment outside of China primarily consist of Managing Your Mood Course, Space from Depression, program NoDep, Well-being course, and Mood GYM, each featuring approximately 5-8 modules, with core content encompassing cognitive restructuring, skill acquisition, and relapse prevention, presented through text, slides, video, or audio formats. Chinese iCBT platforms primarily comprise the 30-day self-help psychological platform and the Chinese version of MoodGYM. As an emerging and efficacious depression treatment modality, iCBT may be employed as a standalone intervention or as an adjunct to other depression treatments^15^.

### 3.1 iCBT Evolution in Recent Years

iCBT represents a novel CBT form that amalgamates traditional Cognitive Behavioral Therapy for Insomnia (CBT-I) with internet technology, thereby transcending temporal and spatial constraints to facilitate convenience and time efficiency. With the internet's rapid expansion, iCBT has experienced commensurate growth. In 2004, Ström et al. pioneered the utilization of an online platform for administering iCBT to insomnia patients^16^. Following a 5-week iCBT course, patients exhibited improvements on par with traditional CBT-I. A recent meta-analysis revealed that iCBT-I can enhance the Insomnia Severity Index (ISI) to a degree comparable to face-to-face CBT-I^17^. Research has indicated iCBT's efficacy extends beyond insomnia treatment, encompassing anxiety, depression, asthma, hypertension, and other conditions^18^.

### 3.2 iCBT Advantages

Relative to traditional face-to-face CBT, Internet-Based Cognitive Behavioral Therapy (iCBT) affords several advantages^19^ by delivering therapy modules online: 1) Enhanced patient accessibility and preservation of medical resources for individuals necessitating intensive care. 2) Standardized treatment provision irrespective of temporal, geographical, and economic constraints. 3) A more confidential platform for patients deterred from face-to-face therapy due to embarrassment, thus encouraging proactive treatment-seeking behavior. 4) Inclusion of additional medical staff in the therapeutic process to alleviate the chief psychotherapist's workload and standardize treatment procedures. 5) Facilitation of medical staff's capacity to monitor patient progress and adjust treatment plans as needed. 6) Timely feedback provision, enabling patients to comprehend their therapeutic journey and exhibit increased compliance^20^.

### 3.3 Advancements and Novel Developments in iCBT

In recent years, the field of iCBT has undergone significant advancements and developments that have transformed the landscape of mental health treatment. With the widespread use of mobile devices, mobile-based iCBT has emerged as an accessible and convenient mode of therapy delivery. Furthermore, the integration of virtual reality (VR) technology has created immersive environments for the treatment of conditions such as social anxiety disorder (SAD) and PTSD. Tailored iCBT, a personalized form of therapy that is customized to meet an individual's unique needs and preferences, has been developed to improve engagement and treatment outcomes.

The integration of iCBT with other technologies has opened up new avenues for treatment. Wearable devices can collect physiological and behavioral data that can provide personalized feedback and insights to users. Chatbots can offer support and guidance to users outside of therapy sessions, while artificial intelligence (AI) can personalize therapy by predicting treatment response and tailoring treatment to individual needs. Blended iCBT, which combines iCBT with face-to-face therapy, has emerged as a hybrid model that leverages the benefits of both modes of therapy. Finally, the choice between guided and unguided iCBT has emerged as a critical consideration, with research suggesting that guided iCBT may be more effective for depression and anxiety. We listed some specific novel approaches and recent advancements and developments in iCBT in **Table 2**, which offer a promising future for the treatment of mental health conditions.

With the power of technology and the principles of cognitive behavioral therapy, iCBT has the potential to provide personalized, accessible, and effective therapy to a wider range of individuals. These innovative therapies have the potential to revolutionize mental health care and help individuals overcome the challenges associated with mental health conditions.

## 4. iCBT Treatment Modalities

iCBT for insomnia encompasses therapist-guided, fully self-help, and semi self-help approaches, which vary based on the degree of therapist involvement. All three modalities have demonstrated efficacy in treating insomnia^33^.

### 4.1 Therapist-Guided iCBT

Therapist-guided iCBT entails patients receiving personalized remote guidance from therapists through voice and video calls, emails, or other communication channels. Therapists can monitor patients' treatment progress and real-time sleep status via the internet, provide guidance for completing online sleep diaries and follow-up questionnaires, and offer timely feedback. Gehrman et al. administered CBT-I to veterans through remote video calls, with participants receiving online treatment six times a week under therapist guidance and completing the ISI questionnaire and sleep diary^34^. The study's findings revealed significant improvements in ISI and all sleep diary parameters except total sleep time, informing the development of the country's clinical telehealth program^34^.

### 4.2 Fully Self-Help iCBT

Fully self-help iCBT enables insomnia patients to independently learn CBT-I-related content through professional platforms featuring e-books, health education booklets, video tutorials, relaxation audios, and more. A recent meta-analysis indicated that self-help CBT-I is as effective as face-to-face CBT for insomnia^35^. Self-help platforms automatically configure the learning process and provide online feedback based on patients' progress. Current self-help platforms include Sleep Healthy Using the Internet (SHUTI) programs, Go! to sleep, CBT-I Coach, Sleepio, Sleepcare, while Chinese self-help platforms feature CCBT, Good Sleep 365 APPs, and related WeChat official accounts such as Accompany You To Sleep.

### 4.3 Semi Self-Help iCBT

Distinct from fully self-help iCBT, semi self-help iCBT involves therapist participation. Therapists develop personalized iCBT programs tailored to patients' specific needs. Should patients encounter questions during self-help iCBT, they can contact their therapists through online platforms, emails, phone calls, or other means. A study involving 118 adult insomnia patients revealed that semi self-help iCBT with partial therapist involvement yielded minimal adverse effects on sleep latency and number of awakenings while significantly enhancing sleep quality, sleep efficiency, and patients' anxiety and depression levels^36^. Compared to the waitlist control group, the semi self-help group exhibited notable improvements in treatment adherence.

## 5. iCBT Platforms

Currently, international online self-help platforms for insomnia encompass Sleep Healthy Using the Internet (SHUTI), Go! to sleep (GTS), CBT-I Coach, Sleepio, and Sleepcare. Domestic platforms include CCBT, Good Sleep 365 APPs, and WeChat official accounts such as Accompany You To Sleep.

SHUTI is a fully automated self-help program designed for adults experiencing chronic insomnia^18^. It comprises sleep restriction, stimulus control, cognitive restructuring, sleep hygiene, and relapse prevention. Each core segment requires approximately 45 to 60 minutes to complete, with the subsequent segment becoming available seven days after the previous segment's completion^18^. A Norwegian study involving 181 participants assigned to either the internet-based CBT-I procedure (SHUTI) group or the internet-based patient education (PE) group demonstrated significantly improved sleep in the SHUTI group compared to the PE group^37^.

GTS is a six-week, interactive online CBT program offered by the Cleveland Clinic Health System for staff and patients^38^. GTS addresses factors impacting sleep quality in insomnia patients by teaching cognitive behavioral techniques and promoting healthy sleep habits to reduce insomnia arising from mental stress and physiological arousal. Bernstein AM et al. utilized the Go! To Sleep program to treat 88 adults with primary insomnia and 263 adults with comorbid insomnia^38^. Post-intervention, both groups exhibited decreased insomnia severity scores and improved sleep quality, with effects persisting through the four-month follow-up^38^.

CBT-I Coach, a product of collaboration between the Virginia National Center for PTSD, Stanford University School of Medicine, and the Department of Defense National Center for Telehealth and Technology, primarily supports patients undergoing CBT-I. Coach provides comprehensive sleep health education, cognitive restructuring, stimulus control, sleep restriction, and relapse prevention. Eric Kuhn et al. implemented CBT-I Coach in veterans with insomnia in the United States, demonstrating the mobile app's capacity to enhance CBT for insomnia^39^.

Sleepio is a fully automated, web-based, self-help sleep intervention program studied in adults with insomnia^40^. Accessible through a browser on a tablet, desktop, or mobile phone, Sleepio is grounded in CBT-I and consists of three components: cognition (paradoxical intention, cognitive restructuring, mindfulness, positive imagery), behavior (sleep restriction, stimulus control, relaxation), and education (sleep hygiene, sleep process). Participants receive six treatments, each lasting approximately 20 minutes, on a weekly basis.

Sleepcare is a fully automated mobile app for insomnia treatment^41^ that includes an online sleep diary, relaxation exercises, sleep restriction exercises, sleep hygiene education, and lasts for 6-7 weeks. Corine HG Horsch et al. demonstrated that patients receiving Sleepcare treatment exhibited lower insomnia severity and improved sleep efficiency compared to a waitlist control group, validating the efficacy of CBT-I delivered through a mobile phone application for insomnia treatment^41^.

## 6. Exploiting the Potential of iCBT to Enhance the Psychological Fortitude of Oncological Patients

### 6.1 Attenuating Adverse Emotional States

#### 6.1.1 Oncological Patients

Surveys intimate that 30-40% of advanced cancer patients experience anxiety, which substantially undermines their quality of life^42^. An alarming 53.7% of malignant tumor patients harbor suicidal ideation, with an even higher prevalence among those afflicted with severe depression^43^. Fear of the disease appears to be the primary catalyst for negative emotions, and emotional disorders such as anxiety and depression frequently contribute to a decline in patients' quality of life. Effectual intervention measures are paramount in mitigating patients' adverse emotions. Fan Gaiping and colleagues allocated 110 laryngeal cancer patients into a control group, which received standard nursing care, and an intervention group, which received, in addition to standard nursing care, one-to-one CBT every other day from 3 days pre-surgery to the 10th day post-surgery, with each session lasting 30 minutes^44^. The study's findings revealed a significantly lower incidence of anxiety in the intervention group compared to the control group (P<0.05), indicating the efficacy of CBT in ameliorating anxiety^44^. Jarzemski et al. discovered that group CBT for prostate cancer patients also considerably alleviated their anxiety and depression^45^. Due to varying sample sizes and assessment tools among studies, the conclusions regarding CBT's effectiveness on psychological symptoms are neither uniform nor exhaustive. To rectify this discrepancy, Ye et al. synthesized recent randomized controlled studies on CBT in breast cancer patients, uncovering that CBT served as an efficacious supplement to conventional treatment in diminishing depression among breast cancer patients, potentially reducing the reliance on antidepressants^46^. Furthermore, researchers determined that CBT might aid patients in attenuating negative psychological symptoms by lessening rumination on past events and apprehension about the future^46^, this meta-analysis solely incorporated women; as men and women exhibit divergent responses to physical and psychological stress, the research conclusions may not be generalizable to men^46^. Consequently, future research should endeavor to conduct evidence-based clinical studies encompassing both genders.

#### 6.1.2 Caregivers of Oncological Patients

Cancer imposes a substantial burden on both patients and their caregivers. Research indicates that caregivers experience elevated levels of anxiety and depression compared to the patients themselves (P<0.01)^47^. Thus, healthcare professionals should also address the negative psychological states of cancer patients' caregivers. Ljungman et al. executed individualized face-to-face CBT for parents of 15 pediatric cancer patients following treatment and assessed their distress levels using self-reported psychological distress scales (including post-traumatic stress symptoms, depression, and anxiety) and the diagnostic MINI-International Neuropsychiatric Interview at baseline, post-CBT, and at the 3-month follow-up^48^. Parents reported significant improvements in PTSD symptoms, depression, and anxiety after CBT compared to baseline. Woodford et al. also observed that internet-based self-help CBT reduced the negative emotions of caregivers of pediatric cancer patients, implying that CBT exhibits a certain efficacy in ameliorating the psychological distress of cancer caregivers^49^.

### 6.2 Rectifying Sleep Disturbances

Although the majority of cancer patients eventually acclimate to their condition, insomnia persists as a significant, yet frequently disregarded, complication of cancer that severely impacts patients' quality of life. Johnson et al. conducted a meta-analysis on the effects of CBT for insomnia (CBT-I) on the sleep status of cancer patients^50^. The results demonstrated that CBT-I effectively enhanced patients' sleep efficiency, minimized wakefulness, and reduced sleep latency. These effects were observed across all cancer patients, irrespective of surgical procedures and disease progression^50^. Garland et al. assessed the efficacy of CBT-I and armodafinil in 88 cancer patients with insomnia^51^. Participants were randomly assigned to one of four groups: armodafinil plus CBT-I, placebo plus CBT-I, armodafinil alone, or placebo alone^51^. CBT-I consisted of seven one-to-one sessions within a week (including three face-to-face interactions and four phone calls), while armodafinil (or placebo) was administered at 50 mg, twice daily^51^. The study found that CBT-I significantly reduced wakefulness and sleep latency in patients, whereas armodafinil did not yield additional benefits in sleep continuity or daytime sleepiness^51^. Moreover, research has shown that, compared to armodafinil, CBT-I improved patients' quality of life by ameliorating insomnia, with such enhancements persisting for at least three months^52^. The beneficial impact of CBT-I for cancer patients with insomnia is robust and enduring. Three years later, Garland et al. compared acupuncture and CBT-I in cancer patients experiencing insomnia symptoms^53^. Patients were divided into two groups and received acupuncture or CBT-I for eight weeks, followed by a 20-week follow-up^53^. Both methods generated meaningful and lasting improvements. Liou et al. obtained similar results in a randomized controlled trial^54^. The authors also discovered that CBT-I was more effective in improving insomnia and fatigue in these patients, especially among those who were white, male, highly educated, and experienced no pain at baseline^54^. In conclusion, CBT exhibits a potent and persistent effect on insomnia in cancer patients, representing a viable option in conjunction with traditional medical and non-medical therapies.

### 6.3 Alleviating Cancer-Related Fatigue

Cancer-related fatigue is a subjective, chronic sensation of exhaustion correlated with cancer treatment. This fatigue often remains unmitigated by rest or sleep and severely disrupts patients' everyday lives^55^. CBT can ameliorate patients' fatigue symptoms. Li et al. implemented CBT for lung cancer patients during the perioperative period, observing significant improvements in fatigue symptoms compared to the control group at three days post-surgery and one day prior to discharge^56^. Zhu et al. conducted a six-month CBT study on lung cancer patients undergoing chemotherapy, also achieving positive results^57^. Additionally, researchers conducted an exploratory study on 35 breast cancer patients who received radiation therapy and experienced side effects such as fatigue and decreased quality of life^58^. Following six weeks of nurse-guided CBT, patients were better able to manage their fatigue levels and exhibited a higher quality of life compared to the control group^58^. This suggests that CBT can improve cancer patients' fatigue symptoms, regardless of differences in disease progression and treatment methods. CBT not only reduces short-term fatigue in cancer patients but also demonstrates long-term efficacy in several randomized controlled trials^59^. Gielissen et al. found that the beneficial effects of CBT persisted until two years post-therapy^60^. To investigate the long-term efficacy of CBT on fatigue, Janse et al. conducted a long-term follow-up on 583 participants with chronic fatigue syndrome who had received CBT, revealing that the benefits of CBT lasted until 18 months post-therapy, with some patients experiencing only mild fatigue even five years after treatment^61^. Van et al. also discovered that although fatigue levels increased over time, the positive effects of CBT persisted in approximately half of the patients during long-term follow-up (14 years)^62^. Undoubtedly, CBT exerts a positive influence on the initial fatigue experienced by cancer patients; however, fatigue relapses can be influenced by various factors, including tumor-related cytokines and hormonal fluctuations. Consequently, it seems more pragmatic and effective to develop fatigue-targeted interventions for patients suffering from fatigue recurrence.

### 6.4 Mitigating Fear of Cancer Recurrence

Fear of cancer recurrence (FCR) pertains to the apprehension, concern, or worry about cancer returning or progressing^63^. It is among the most frequently cited issues for cancer patients. A survey found that 30% to 70% of cancer patients experience FCR, which has become a chronic problem impacting their quality of life and daily functioning^64^. While a certain degree of fear is acceptable as it fosters vigilance for potential recurrence signs and encourages adherence to regular check-ups and medical protocols, high levels of fear are associated with diminished quality of life, functional impairment, anxiety, depression, excessive comfort-seeking, and increased healthcare costs^65^. Van et al. administered a 12-month CBT program to a breast cancer patient with FCR, encompassing seven face-to-face therapy sessions and one phone therapy session^66^. During treatment, the patient's FCR was reduced and better managed, with improvements still evident at six- and 12-month follow-ups^66^. The patient also demonstrated enhancements in depression and fatigue symptoms at 12 months post-therapy, and their attitude towards life improved compared to pre-therapy^66^. Van et al. performed mixed CBT for 45 patients with high-level FCR between six months and five years post-treatment for prostate, rectal, and breast cancers, finding that CBT could alleviate the severity of patients' FCR^67^. Another randomized controlled trial enrolled patients with severe FCR following treatment for breast, prostate, and colorectal cancer, evaluating the long-term efficacy and cost-effectiveness of CBT. Data were collected at baseline, three months, nine months, and 15 months, with results demonstrating significant improvements in FCR in the CBT group at all time points compared to the control group that received conventional care^68^. Furthermore, CBT incurred lower costs than conventional care, indicating that CBT represents a relatively cost-effective intervention. Studies on FCR in China are limited, and intervention programs for FCR are rarely mentioned in the literature. Additional evidence is anticipated to support the effectiveness of CBT on FCR in cancer patients.

## 7. Quintessential Tenets, Approaches, and Potency of iCBT for Insomniac Patients with Cancer

### 7.1 Foundational Principles of iCBT for Insomnia

Spielman et al. postulated the Spielman tripartite conjecture in 1987, alternatively termed the 3P hypothesis, encompassing Predisposing factors, Precipitating factor, and Perpetuating factors of insomnia^69^. Predisposing factors allude to an individual's gender, age, personality, and genetic traits predisposing them to insomnia; Precipitating factors pertain to recent stressful events potentially engendering acute insomnia; and Perpetuating factors denote maladaptive behaviors and beliefs in response to transient insomnia, such as elongating daytime naps, advancing nocturnal bedtime, and postponing morning rising, thus perpetuating insomnia^69^. Cognitive Behavioral Therapy (CBT) is predicated upon this hypothesis.

### 7.2 iCBT Methodologies for Insomnia

Drawing from the aforementioned theories, Morin et al. advanced Cognitive Behavioral Therapy for insomnia (CBT-I) in 1993, comprising sleep hygiene education, sleep restriction therapy, stimulus control therapy, cognitive therapy, and relaxation therapy^70^. Individuals with chronic insomnia typically receive multiple concurrent therapies, with monotherapy seldom employed. Sleep hygiene education, sleep restriction therapy, and stimulus control therapy are regarded as primary interventions for insomnia, and constitute the indispensable core components of CBT-I combination therapy. In practical implementation, therapists may select therapeutic combinations contingent upon specific circumstances.

### 7.3 Efficacy of iCBT for Insomnia

Since Ström et al. pioneered the utilization of a network platform to administer a 5-week CBT program for insomniacs in 2004, iCBT has been extensively applied in clinical settings, yielding favorable outcomes^16^. A meta-analysis encompassing 11 randomized controlled trials (1,460 participants in total) revealed that online CBT-I ameliorated insomnia severity, sleep efficiency, subjective sleep quality, sleep latency, total sleep time, and diminished nocturnal awakenings, with comparable efficacy to traditional face-to-face CBT-I^17^. Espie et al.'s findings demonstrated the effectiveness of iCBT in mitigating insomnia symptoms and enhancing functional health, mental health, and sleep-related quality of life among insomniac patients^71^.

### 7.4 Potency of iCBT for Insomnia Comorbidities

In the context of insomnia concomitant with mental illness, Espie et al. conducted a comprehensive study involving 3,755 students^41^. The findings indicated that iCBT engendered positive psychological shifts, ameliorating students' insomnia as well as mental health and quality of life. iCBT also demonstrated significant efficacy in addressing insomnia associated with somatic diseases. Patel et al. executed a single-center randomized controlled trial, juxtaposing iCBT and standardized sleep hygiene education in treating Parkinson's disease with concomitant insomnia, discovering greater improvement in insomnia severity scores following iCBT compared to standard education^72^. A Danish investigation enrolled 255 breast cancer patients experiencing clinically significant sleep disturbances, randomly assigning participants to an iCBT group and a waiting group^73^. The results suggested substantial improvement in insomnia severity, sleep quality, and fatigue severity, leading investigators to advocate for the incorporation of iCBT treatment into the National Cancer Rehabilitation Program^73^.

## 8. Current iCBT Implementation in Oncology Patients with Depression

A number of researchers have investigated the mechanism and impact of the Chinese version of MoodGYM in depressive patients, recruiting 62 college students with depression^74^. The outcomes revealed that 52.94% of patients in the iCBT group experienced clinically meaningful changes, while only 9.09% in the control group demonstrated such changes^74^. Subsequently, Xie et al. employed the Chinese version of MoodGYM to ameliorate depression and anxiety among nursing staff, finding that the experimental group exhibited greater improvement in depression, anxiety status, job satisfaction, and subjective well-being compared to the control group^75^. Li Jinyang et al. also researched the application of iCBT in the Chinese population, with results indicating that iCBT improved depressive symptoms in subjects; however, the dropout rate reached 78%^76^. In summary, there is a paucity of research on iCBT application in China. iCBT remains in an exploratory phase, presenting ample opportunities for development. Conversely, iCBT is relatively mature in foreign countries and is widely employed.

### 8.1 iCBT Utilization in Depressed Patients Across Diverse Age Groups

iCBT is applicable to various age demographics, ranging from adolescents to the elderly. Topooco et al. conducted iCBT on 33 adolescent patients with depression^77^. Following iCBT, patients' average Beck Depression Inventory-II (BDI-II) scores diminished from 33.1 to 19.9, and Patient Health Questionnaire-9 (PHQ-9) scores decreased from 15.2 to 9.7^77^. This intervention exerted a significant effect in reducing depressive symptoms among adolescents, and patients' general self-efficacy was also substantially bolstered^77^. The PHQ-9 scores of adult patients with depression similarly declined significantly after iCBT (P<0.05), alleviating depressive symptoms and, more importantly, enhancing patients' responsiveness to clinical treatment^77^, ^78^. Moreover, 29 elderly patients with depression experienced decreased PHQ-9 scores from 11.04 to 3.96 and Generalized Anxiety Disorder-7 (GAD-7) scores from 7.44 to 2.96^79^.

### 8.2 iCBT Deployment in Varied Settings

The application of iCBT is progressively expanding, with implementation in outpatient clinics and primary health care centers yielding favorable therapeutic outcomes. Mathiasen et al. provided iCBT to 60 patients with depression in a Danish outpatient clinic^80^. Post-treatment, the PHQ-9 scores significantly decreased, and efficacy was positively correlated with time spent on the intervention^80^. Additionally, in patients treated with iCBT in a Swedish psychiatric outpatient clinic, Hospital Anxiety and Depression (HADs-D) scores declined, depressive symptoms were alleviated, and such symptom improvement persisted at 6-month and 12-month follow-up evaluations^81^. Canadian authorities encouraged the widespread adoption of iCBT in mental health care centers^82^. Furthermore, scholars in the United Kingdom investigated the effects of iCBT in primary health care centers, discovering no difference in BDI-II scores between patients receiving iCBT and those receiving conventional pharmacotherapy and psychotherapy post-treatment and at one-year follow-up^83^.

### 8.3 iCBT Application in Patients with Differing Depression Severities

A decade ago, iCBT-related research primarily targeted patients with mild to moderate depression, excluding individuals with severe depression and suicidal ideation. In recent years, an increasing number of researchers have investigated the therapeutic impact of iCBT on patients with major depressive disorder and suicidal ideation. Richards et al.^84^ and Mewton et al.^85^ substantiated the therapeutic efficacy of iCBT in patients with major depressive disorder and suicidal ideation, respectively. Given the strong correlation between depression and anxiety, Mathiasen et al. discovered that iCBT can ameliorate symptoms of both depression and anxiety^80^. iCBT is not only effective for treating mild to moderate depression but also demonstrates therapeutic benefits for patients with suicidal ideation, severe depression, and depression accompanied by anxiety.

### 8.4 iCBT Efficacy for Cancer Patients with Depression

#### 8.4.1 Reduction in Suicidal Ideation Occurrence

Depression constitutes a significant risk factor for suicide and suicide-related behaviors. Nevertheless, the majority of iCBT studies excluded patients with suicidal ideation. Mewton et al. examined the impact of iCBT on suicidal ideation in 484 patients with depression, finding that after six online courses, the incidence of suicidal ideation decreased from 50% to 27%^85^. iCBT can significantly reduce suicide rates among patients with depression.

#### 8.4.2 Improvement in Depressive Symptoms and Relapse Prevention

Subclinical depression refers to individuals who exhibit depressive symptoms but do not meet the diagnostic criteria for depression^86^. Subclinical depression is prevalent and associated with diminished quality of life, economic costs, and increased mortality. Earley et al. conducted iCBT on 29 participants with BDI-II scores ranging from 14~28. Post-intervention, participants enhanced their self-efficacy, acquired new skills for managing symptoms and emotions, and reduced their risk of developing major depression. Furthermore, a meta-analysis revealed that iCBT not only significantly improved depressive symptoms in patients with subclinical depression but also enhanced their social functioning^87^. iCBT can aid patients with subclinical depression in improving depressive symptoms and reducing the likelihood of developing major depression, rendering it a valuable intervention to promote.

#### 8.4.3 Resource Conservation and Treatment Cost Reduction

Traditional CBT necessitates professional psychiatrists, whereas iCBT can be implemented by nurses under psychiatrists' supervision after appropriate training, addressing the issue of inadequate medical staff to some extent. iCBT is inherently time-efficient, with therapists providing 10-30 minutes of guidance weekly based on patient responses (10~15 minutes suffices in most cases). Numerous studies have assessed the cost-effectiveness of iCBT for depression treatment. A meta-analysis summarizing research on iCBT cost-effectiveness determined that, from social and health service provider perspectives, iCBT is highly cost-effective^88^.

## 9. Investigations into Genetic Underpinnings of CBT/iCBT Efficacy for Anxiety Disorders

In recent years, there has been a burgeoning interest in examining genetic and biological determinants that contribute to the effectiveness of CBT/iCBT. Understanding the role of genetic factors in predicting CBT/iCBT efficacy could facilitate the development of highly individualized and efficacious treatments by elucidating the psychopathological mechanisms underlying anxiety disorders. Numerous candidate genes have been identified as potential predictors of patients' responsiveness to CBT/iCBT, including FK506 binding protein 5 (FKBP5), monoamine oxidase A (MAOA), catechol-O-methyltransferase (COMT), serotonin transporter (5-HTT), tryptophan hydroxylase (TPH), brain-derived neurotrophic factor (BDNF), and nerve growth factor (NGF).

### 9.1 Influence of HPA Axis-Related Genes on iCBT Efficacy in Treating Anxiety Disorders

FKBP5 is integral to stress responses as it modulates the HPA axis. Upon exposure to a stress-inducing environment, glucocorticoids, such as cortisol, are rapidly secreted. FKBP5 attenuates glucocorticoid responsiveness by reducing receptor affinity and impeding their nuclear translocation^89^. Glucocorticoids govern FKBP5 mRNA expression via their response elements, thereby establishing an ultra-short negative feedback loop. Dysregulation of this process can precipitate disorders such as anxiety^90^.

The impact of FKBP5 on the efficacy of CBT/iCBT for anxiety disorders has been rigorously investigated. Roberts et al. discovered that, among individuals possessing risk alleles, those who exhibited the most significant improvement in symptoms following CBT/iCBT experienced a reduction in FKBP5 intron 7 methylation^91^; however, these findings were incongruous with prior results^91^. Additionally, it was observed that FKBP5 expression levels diminished in patients who demonstrated a superior response to CBT/iCBT^92^. Earlier studies revealed that childhood trauma might induce heightened methylation of FKBP5 intron 7 in patients with risk alleles, potentially serving as a connection between childhood trauma and an enhanced response to CBT/iCBT. The current research indicates that FKBP5 may affect CBT/iCBT efficacy in patients with anxiety disorders, though the specific genetic mechanism remains to be elucidated. Future studies could adopt an integrative approach, considering genotype, DNA methylation, and gene expression when examining the influence of this gene on iCBT efficacy in treating anxiety disorders.

### 9.2 Implications of 5-HT System-Related Genes on iCBT Efficacy in Treating Anxiety Disorders

#### 9.2.1 Serotonin Transporter (5-HTT)

The 5-HTT protein is a constituent of serotonergic nerve terminals and functions to reuptake 5-HT from the synaptic cleft into synaptic neurons, thereby regulating the concentration of 5-HT in the synaptic cleft. The 5-HTT-linked polymorphic region (5-HTTLPR) is situated approximately 1 kb upstream of the transcription start site of the 5-HTT gene. The 5-HTT alleles can be classified as short (S) or long (L) based on the absence or presence of a 43-bp insertion at the 5-HTTLPR, with the S allele being correlated with lower 5-HTT expression and slower 5-HT uptake^93^.

Research has demonstrated that dysregulation of the 5-HT neurotransmitter system is linked to anxiety susceptibility, as specific selective 5-HT reuptake inhibitors exhibit efficacy for anxiety disorders, suggesting a role for this system in the path ological mechanism of anxiety^94^. Alterations in emotion, behavior, and cognition following CBT/iCBT are connected to metabolic changes within associated brain regions^95^. Among children with anxiety disorders and panic disorders with agoraphobia (PDA), those possessing the SS genotype exhibited a superior response to CBT/iCBT compared to individuals with the LL or SL genotype^96^. However, the influence of 5-HTTLPR on CBT/iCBT efficacy was not observed in two other studies focusing on SAD^97^, ^98^. From an epigenetic standpoint, methylation of a CpG site within the promoter region of the 5-HTT gene was more frequently observed in children with primary anxiety disorder who responded favorably to CBT/iCBT^99^. A radio-genomic study did not find an effect of 5-HTTLPR on the response of PDA patients to CBT/iCBT, but it did observe negative anterior cingulate cortex-amygdala coupling in responders with the LL genotype^100^. It is hypothesized that the S allele possesses lower transcription efficiency, and methylation further suppresses 5-HTT transcription, leading to a decrease in 5-HTT expression and 5-HT reuptake. The accumulation of 5-HT results in heightened amygdala reactivity, which is associated with increased fear^101^. Consequently, the S allele is correlated with a high response, meaning that patients carrying this allele are more susceptible to mental disorders in adverse environments, while positive environmental factors may confer greater benefits. The precise mechanism through which 5-HTTLPR genotype and its methylation impact CBT/iCBT for anxiety disorders remains to be clarified; nevertheless, future studies investigating CBT/iCBT efficacy for anxiety disorders should consider 5-HTTLPR genotype, methylation, and expression.

#### 9.2.2 Tryptophan Hydroxylase (TPH)

TPH constitutes the rate-limiting enzyme in 5-HT synthesis and comprises two isoforms: TPH1 and TPH2. TPH1 is localized to chromosome 11p15.3-11p14, whereas TPH2 is situated on chromosome 12q21 and exhibits neuron-specificity (predominantly expressed in the brain)^102^.

The G703T polymorphism within the TPH2 promoter region has been associated with amygdala hyperresponsiveness and regulation of fear. Carriers of the T allele displayed greater improvement in social anxiety symptoms following CBT/iCBT, while patients with the GG genotype exhibited a superior response during the initial 9 weeks of CBT/iCBT^98^. Given that amygdala response to emotional stimuli is modulated by 5-HT^103^ and TPH2 is responsible for 5-HT synthesis, it is highly plausible that the G allele at this polymorphic site correlates with a diminished response to CBT/iCBT via reduced 5-HT synthesis. Conversely, Furmark et al. discovered that the social anxiety symptoms of patients with the GG genotype improved more than those of T allele carriers after treatment^104^. The discrepancies between these two studies may be attributable to variations in study location and duration, as well as therapy course. Additionally, the small sample size could hinder the detection of subtle genetic effects. Future investigations might consider utilizing larger sample sizes.

### 9.3 Implications of Monoamine Oxidase System Genes on iCBT Efficacy for Anxiety Disorders

#### 9.3.1 Catechol-O-Methyltransferase (COMT)

COMT is an enzyme responsible for the methylation and degradation of monoamine neurotransmitters, such as dopamine^105^. A substitution of G1947 by A in the third exon of the COMT gene results in the replacement of Val158 by Met, consequently decreasing enzyme activity. Consequently, carriers of the Met allele at this site exhibit higher synaptic dopamine levels^106^, leading to the consolidation of fear memory^107^.

Research has demonstrated that patients who have experienced childhood trauma exhibit increased fear when carrying a Met/Met genotype, and the Met allele is also associated with negative emotions and alterations in prefrontal cortex activity^108^. In several case-control studies, panic disorder (PD) patients with the Met/Met genotype were found to be less responsive to CBT/iCBT compared to G allele carriers^109^. However, the results were not entirely consistent; for instance, Hedman et al. discovered that COMT polymorphisms did not affect CBT/iCBT efficacy in SAD patients^97^. Potential mechanisms may include a fear network associated with amygdala hypersensitivity, which causes panic attacks^110^. Medial and ventral prefrontal regions can regulate the amygdala, while the COMT val158met polymorphism influences prefrontal dopamine levels and prefrontal cortex function, subsequently modulating emotional response. In patients with the Met/Met genotype, cognitive control of emotional responses may be impaired, resulting in reduced CBT/iCBT efficacy. The aforementioned studies displayed inconsistencies regarding the impact of COMT polymorphisms on CBT/iCBT efficacy, which may be due to different anxiety disorder subtypes. Future studies may consider larger sample sizes encompassing multiple anxiety disorder subtypes.

#### 9.3.2 Monoamine Oxidase A (MAOA)

MAOA, encoded by the MAOA gene at Xp11.4-p11.3, primarily participates in the degradation of 5-HT, norepinephrine, and dopamine. The MAOA variable nucleotide tandem repeat (MAOA-VNTR) consists of 30-base repeating fragments and is located upstream of the MAOA gene promoter. Prior research has indicated that this gene polymorphism is associated with various psychiatric disorders, including anxiety disorders^111^. Reif et al. found that PD patients carrying one or two long high-activity MAOA-VNTR alleles exhibited a 46% response rate to CBT, which was significantly lower than the 67% response rate observed in patients homozygous for the short MAOA-VNTR allele^112^. A potential mechanism is that patients with short low-activity MAOA-VNTR alleles display increased anterior cingulate cortex responses to threatening stimuli, which are related to emotional processes. CBT/iCBT modifies the brain's processing of threats, augmenting parietal responses, which are associated with perceptual processes, and shifting from emotional to perceptual processes during cognitive-behavioral testing. This change in cognition towards a potentially threatening stimulus alters the individual's response (“do not evade”). From an epigenetic perspective, it was discovered that MAOA methylation significantly increased in CBT responders to levels comparable to those of healthy controls^113^. It is postulated that demethylation of the MAOA gene may result in the activation of MAOA expression, consequently decreasing the concentration of monoamines in the synaptic cleft and increasing the risk of PD. Research has demonstrated that both MAOA gene polymorphism and methylation contribute to the efficacy of CBT/iCBT in the treatment of anxiety disorders. However, the current body of genetic research primarily focuses on panic disorder, and the impact of MAOA on the effectiveness of CBT for other subtypes of anxiety disorders remains unexplored.

Future studies should aim to elucidate the role of MAOA gene polymorphism and methylation in the efficacy of CBT/iCBT for various anxiety disorder subtypes. Such investigations should consider larger sample sizes and diverse anxiety disorder subtypes to provide a comprehensive understanding of the influence of MAOA on CBT's effectiveness across different manifestations of anxiety disorders.

### 9.4 Interrelation Between Neurodevelopment-Associated Genes and Potency of iCBT in Alleviating Anxiety Disorders

Neurotrophic factors constitute a class of growth factors that exert a direct influence on neuronal cells, fostering their growth, differentiation, and survival. These factors exhibit widespread expression within the limbic region of the central nervous system, an area intimately connected to mood and cognitive functionality. Investigations exploring the association between neurotrophic factors and the efficacy of iCBT in ameliorating anxiety disorders have predominantly concentrated on BDNF and NGF.

BDNF serves as a crucial neural growth factor implicated in the development and plasticity of the nervous system, as well as in modulating various neurotransmitters such as 5-HT. The Val66Met single nucleotide polymorphism (SNP) of BDNF impacts its intracellular transport and secretion, resulting in diminished BDNF secretion in Met carriers^114^. NGF, on the other hand, influences synaptic plasticity, neurogenesis, and the endocrine regulation of stress responses. It acts upon axonal growth, inducing structural alterations in specific neural circuits^115^.

Stressful environments can alter the expression of central BDNF, and the serum BDNF levels in patients with panic disorder (PD) are lower than those observed in healthy controls^116^. A recent study discovered that methylation at the BDNF promoter region might be associated with depression in the context of PD but does not influence the effectiveness of CBT/iCBT in PD patients^114^. Elevated serum NGF serves as an indicator of positive response to CBT/iCBT in individuals with generalized anxiety disorder (GAD)^117^. Lester et al. discovered that the NGF rs6330 T allele appears to correlate with favorable response to CBT/iCBT in children afflicted by anxiety disorders^118^ ^106^. This effect demonstrated a cumulative nature, with increased T alleles corresponding to enhanced efficacy. It has been conjectured that children carrying the T allele may exhibit subtle variations in neurotrophic signaling, potentially resulting in alterations in neuroplasticity in response to environmental stimuli and, consequently, impacting mood regulation. Existing research indicates that neurotrophic factors somewhat influence the efficacy of CBT/iCBT for anxiety disorders, but only genetic polymorphisms of these factors have been examined^118^. In the future, the epigenetics of neurotrophic factors should be evaluated as potential predictors of CBT/iCBT efficacy in the treatment of anxiety disorders.

### 9.5 Influence of Endocannabinoid System-Related Genes on the Endocrine Efficacy of iCBT in Addressing Anxiety Disorders

The endocannabinoid (ECB) system comprises cannabinoid receptors, endocannabinoids, and catabolic enzymes responsible for the degradation of endocannabinoids. A multitude of studies have implicated endocannabinoids in anxiety, fear extinction, and emotional processing^119^. Fear memory extinction constitutes a crucial component of CBT/iCBT, characterized by patients' defensive responses gradually diminishing following repeated exposure to stimuli associated with fear memories but lacking harmful consequences. Roberts et al. discovered that the expression level of the diacylglycerol lipase β gene, involved in the biosynthesis of 2-AG (an endocannabinoid), correlated with the extent of anxiety reduction following CBT^92^. The rs12133557 and rs6928813 single nucleotide polymorphisms (SNPs) of the cannabinoid receptor gene predicted enhanced CBT/iCBT efficacy^120^. Cannabinoid receptors are highly expressed in brain regions implicated in fear processing (such as the amygdala, hippocampus, and medial prefrontal cortex), enabling cannabinoids to influence neurotransmitters involved in anxiety (including gamma-aminobutyric acid, glutamate, and serotonin). It has been postulated that these SNPs may augment the ECB system's effect and thereby regulate anxiety. These findings unequivocally demonstrate the ECB system's impact on CBT/iCBT's functional mechanisms, suggesting a potential novel target for addressing anxiety disorders. Nonetheless, the molecular mechanism through which this system's genes affect anxiety disorders has not been thoroughly investigated. Future research should elucidate the molecular pathways by which gene polymorphisms impact endocannabinoid receptor function.

### 9.6 Additional Genes Affecting iCBT Efficacy in the Treatment of Anxiety Disorders

Various other genes have been implicated in the efficacy of CBT/iCBT in treating anxiety disorders, such as Glutamate Receptor, Ionotropic Kainate 4 (GRIK4), Hypocretin/hypocretin receptor 1 (HCRTR1), and Interleukin 1 Receptor Type 1 (IL1R1). Researchers observed that GRIK4 rs1954787 influences CBT/iCBT efficacy in children with anxiety disorders, HCRTR1 rs2271933 affects CBT/iCBT efficacy in panic disorder/agoraphobia (PD/AG) patients^121^, and methylation of IL1R1 cg06943668 increases following CBT/iCBT in PD patients^122^.

## 10. Unique Implementations of iCBT for Breast Cancer Patients

### 10.1 Enhancement of Sexual Function

Hummel et al. (2015) devised an iCBT intervention targeting sexual dysfunction in breast cancer patients^123^, which was subsequently refined^124^. The researchers executed a 24-week randomized controlled trial, involving 169 breast cancer patients exhibiting sexual dysfunction, sourced from 10 hospitals in the Netherlands^124^. Participants in the intervention group engaged in iCBT sessions, centered on “ameliorating sexual and intimacy issues,” for durations of 90-120 minutes weekly during the trial^124^. The intervention encompassed the following facets: 1) Therapists: A consortium of 3 proficient sexologists and a psychologist convened weekly to assess the patient's extant treatment strategy and recommend modifications. 2) Structure: A psychologist or sexologist spearheaded the group, selecting 4 to 5 germane treatment modules for the patient based on their specific circumstances; the therapist provided weekly guidance via email, with voice calls at the 10th and 24th weeks. 3) Treatment adherence: To bolster patient compliance, therapists offered encouragement at the 12th and 18th weeks. 4) Spousal involvement (non-compulsory): The therapist apprised the patient of the significance and supplementary benefits of spousal participation prior to protocol implementation. 5) Internet platform: Patients accessed the website using personal login credentials. 6) Project classification: Philanthropic initiatives. 7) Therapeutic modules: Ten in total, encompassing problem identification, sexual relationship evaluation, sex and body image, concentration, bodily exploration, sexual desire (male partner), sexual desire (female partner), cognitive alterations, sexual orientation, and relapse prevention^124^. Each module comprised informative texts and relevant interventions, partitioned into four segments: introduction, psychoeducation, assignments, and feedback. Patients were required to finalize the current module before progressing to the subsequent one. The control group received informational brochures addressing coping strategies for sex and cancer concerns. The investigators employed the Female Sexual Function Index Questionnaire, the Sexual Behavior Questionnaire, and the modified version of the Female Sexual Distress Scale to assess the sexual function of both groups^124^.

Following 12 weeks of intervention, the experimental cohort demonstrated substantially elevated sexual desire and satisfaction, as well as reduced sexual discomfort relative to the control group. After 24 weeks, 63.2% of patients in the experimental group exhibited enhanced sexual function, compared to only 32.0% in the control group, with the overall sexual function improvement in the experimental cohort being 3.66 times that of the control group. Reevaluation of the experimental group's sexual function at the 3rd and 9th months post-intervention revealed the enduring efficacy of iCBT in bolstering patients' sexual function^125^. Moreover, the study identified that spousal participation and high patient compliance during iCBT implementation could augment its efficacy in improving patients' sexual function^125^. However, the researchers did not query the patients regarding their preferences when choosing treatment modules, and treatment adherence and follow-up rates were suboptimal (62%, 82%). Additionally, the therapists did not personally conduct patient follow-ups. Future research should address these concerns^125^.

### 10.2 Alleviation of Menopausal Symptoms

Atema et al. formulated an iCBT intervention aimed at ameliorating menopausal syndrome in breast cancer patients, with particular focus on hot flushes and night sweats (HFNS) symptoms, stress, and sleep disturbances^126^. The team conducted a randomized controlled trial involving 254 breast cancer patients with menopausal syndrome, recruited from 12 hospitals in the Netherlands^127^. The patients were allocated into two groups: an experimental group receiving iCBT and a control group receiving standard care, each for 6 weeks, with follow-ups at the 10th and 24th weeks post-intervention. The iCBT program for menopausal syndrome in breast cancer patients comprised 6 treatment modules: introduction, hot flash reduction, stress relief, sleep enhancement, body image improvement and sexuality, reflection, and maintenance plan formulation. Each module consisted of self-reflection, phase-specific training plans, psychoeducation, and assignments, requiring approximately 60 minutes to complete (with 30 minutes allocated for assignments). Based on the patients' submitted assignments, therapists provided pertinent feedback via email on a weekly basis. Simultaneously, the experimental group was subdivided into two subgroups according to iCBT implementation: the guided iCBT group and the self-help iCBT group. Compared to the self-help group, the guided iCBT group participated in a 30-minute telephonic consultation with the therapist prior to the intervention, could only advance to the subsequent module upon reading the feedback email, and had access to the therapist's support via email at any time. During follow-ups, the HFNS frequency and overall severity of menopausal symptoms were assessed using the Hot Flash Problem Rating Scale and the Functional Assessment of Cancer Therapy (FACT) - Endocrine Symptoms Subscale^127^.

This series of studies discovered that: 1) iCBT effectively mitigated the cognition and frequency of HFNS and the overall severity of menopausal symptoms in 82.6% of breast cancer patients, with enduring effects^127^; 2) Guided iCBT exhibited superior compliance and satisfaction (≥90%) compared to self-administered iCBT^128^; 3) Patients with lower educational attainment reaped the most significant benefits from iCBT, and healthy HFNS beliefs contributed to iCBT's efficacy in enhancing patients' menopausal symptom management^129^. However, the researchers did not evaluate the efficacy immediately after the intervention, and the sample size of each study group was limited. Presently, few clinical trials have explored this area, necessitating future multi-center, large-sample, high-quality randomized controlled trials to further corroborate the efficacy of iCBT in addressing menopausal syndrome in breast cancer patients. Verbeek et al.^130^ conducted a cost-effectiveness analysis based on the studies by Atema et al.^126^-^129^, concluding that both iCBT variants were cost-effective, with self-administered iCBT offering greater benefits and minimal impact on healthcare budgets^130^. Further research should explore methods to enhance self-management awareness and capabilities in patients undergoing self-help iCBT.

### 10.3 Enhancing Sleep Quality

Presently, two principal varieties of iCBT address insomnia symptoms in breast cancer patients: iCBT for Insomnia (iCBT-I) and Fully Automated Interactive iCBT (also known as SHUTi)^131^. iCBT-I, a website devised by Dozeman et al., caters specifically to breast cancer patients experiencing insomnia symptoms^132^. Comprising six treatment modules, it encompasses themes such as psychoeducation concerning sleep and insomnia, sleep hygiene and behavior, initiating a novel sleep cycle, relaxation techniques, contemplations on sleep dysfunction, and relapse prevention^132^. A nine-week study involving 171 participants with insomnia symptoms from three Dutch oncology hospitals assessed the application of iCBT-I in breast cancer patients^132^. Researchers evaluated insomnia and fatigue severity and determined whether patients' anxiety and depressive symptoms ameliorated using the Hospital Anxiety and Depression Scale^132^. The findings demonstrated that iCBT-I substantially improved insomnia, fatigue, anxiety, and depression symptoms in breast cancer patients, with younger patients and those suffering from severe insomnia deriving greater benefits^132^. However, the intervention frequency remains indeterminate, subject inclusion criteria were not stringent (e.g., patients were included based on self-reported insomnia symptoms without clinical validation), and the absence of a control group necessitates further verification of its efficacy^132^.

SHUTi is a structured program designed for adult insomnia treatment, encompassing six treatment modules: introduction to treatment principles, sleep restriction, stimulus control, cognitive reconstruction, sleep hygiene, and relapse prevention^73^. Zachariae et al. conducted a 15-week randomized controlled trial with 255 Danish breast cancer patients experiencing insomnia symptoms^128^. The experimental group received SHUTi, comprising nine weeks of treatment and six weeks of follow-up, with each module lasting 45-60 minutes, whereas the control group received no specific treatment^128^. Researchers assessed insomnia severity using the Insomnia Severity Index Questionnaire, as well as fatigue severity. After the nine-week intervention, 60.5% of the experimental group exhibited significant improvements in insomnia symptoms, 31.8% in sleep quality, and 52.1% in sleep efficiency, while the control group's rates were 14.2%, 5.0%, and 12.3%, respectively^16^. Additionally, the study found sustained improvements in insomnia and fatigue symptoms in breast cancer patients following SHUTi^80^. However, the control group received no specific treatment, and the efficacy of SHUTi was analogous to that of traditional face-to-face CBT in cancer patients^50^, warranting further validation of SHUTi's efficacy in future research.

### 10.4 Alleviating Fatigue

“Road to Recovery” is an iCBT platform that concentrates on breast cancer patients' fatigue symptoms^59^. Abrahams et al. conducted a six-month randomized controlled trial involving 132 severely fatigued breast cancer patients from eight Dutch hospitals^9^. The experimental group received therapy via “Road to Recovery” twice weekly, including eight treatment modules addressing goal setting, coping with breast cancer and anti-cancer treatment, managing fear of cancer recurrence, fostering empathy, adjusting sleep-wake rhythms, modifying activity levels, enhancing social support, and assessing goal attainment^59^. Except for “Setting goals” and “Goal attainment,” the remaining modules each contain three components: psychoeducation (“reading”), fatigue-reduction measures (“doing”), and patient self-evaluation (“reviewing”). Additionally, therapists were required to provide feedback on treatment implementation and address patient inquiries via email or video conference sessions twice weekly. The control group received standard care^59^. Researchers assessed fatigue, quality of life, anxiety, and depression in both groups using the Fatigue Severity Scale in the Personal Intensity Inventory, the European Organization for Cancer Research and Treatment Quality of Life Scale, and the Brief Symptom Scale. The results revealed that, compared to routine care, iCBT improved fatigue symptoms in 73% of patients^59^. Concurrently, iCBT significantly reduced anxiety and depression symptoms in breast cancer patients and enhanced their quality of life. However, the lengthy treatment duration potentially resulted in suboptimal patient compliance, and the high therapist workload may have adversely impacted treatment efficacy. Future in-depth investigations should explore methods to abbreviate the treatment duration and employ AI-assisted feedback to address these issues without diminishing iCBT's effectiveness.

## 11. Profits and Constraints of iCBT

### 11.1 Profits of iCBT

iCBT possesses the subsequent advantages: 1) Alleviating therapist scarcity: Conventional CBT-I necessitates therapists to engage in face-to-face, individualized interactions with patients. Nevertheless, the substantial quantity of insomnia sufferers surpasses the considerably smaller populace of sleep behavioral therapists. iCBT provides CBT-I via the internet to counteract this issue. 2) Unrestricted by temporal and spatial constraints: Conventional CBT mandates that both therapists and patients coexist within a designated environment at a predetermined time. iCBT offers enhanced flexibility in terms of time and space, catering to the patient's requirements. For individuals necessitating treatment in remote locations, iCBT is undeniably the optimal selection. 3) Reduced expenditures: iCBT conserves considerable time for therapists, augments treatment efficiency, and diminishes treatment expenses. 4) Enhanced flexibility: Patients can liaise with therapists on internet platforms, thereby amplifying the adaptability of the treatment process.

### 11.2 Constraints of iCBT

iCBT is encumbered by the ensuing limitations: 1)Researchers lack comprehensive understanding of each patient's insomnia causality and cannot thoroughly ascertain adherence to the treatment plan. 2) Compliance rates for iCBT programs are deficient. Patel et al.'s investigation demonstrated that a mere 57% of patients within the iCBT cohort completed the treatment regimen^72^. 3) iCBT implementation typically necessitates patients to generate subjective sleep records on online platforms; the absence of objective sleep monitoring renders accurate evaluation of patients' sleep conditions arduous. 4) iCBT presently exhibits minimal concern for patient safety and lacks suitable evaluation and warning systems for patients at risk of self-harm or suicide.

#### 11.2.1 Elevated Attrition Rate and Suboptimal Compliance

iCBT courses exhibit standardized content and structure, potentially hindering therapists from delivering timely feedback and adjusting the program in accordance with the patient's response, which consequently impairs patient compliance. Li Jinyang et al. discovered that iCBT treatment attrition rates reached 78%, in contrast to the mere 11% observed in a randomized clinical controlled trial^76^.

#### 11.2.2 Intricate Challenges in Practical Implementation

Five European nations have enacted iCBT, yet each has confronted the subsequent conundrums: how to assimilate it into the mental health care system, how to enlist patients, how to guarantee therapist work quality, and how to furnish long-lasting, sustainable iCBT treatment^133^. Concurrently, the establishment of pertinent infrastructure necessitates substantial human, material, and financial resources, and patient data security and privacy present certain risks. These factors hinder iCBT implementation and negatively impact long-term therapeutic results. Unraveling the intricacies of iCBT functionality and surmounting the obstacles to implementation are pivotal to further inquiry within this domain.

#### 11.2.3 Ambiguous Influential Factors

Both patient and therapist behaviors during treatment can critically impact the outcome. Presently, research addressing factors influencing treatment results is scarce. One study scrutinized influential factors based on email correspondence between patients and therapists, identifying therapist affirmation, encouragement, and self-disclosure as positively impacting treatment outcomes^134^. In future iCBT implementation, such constructive behavior ought to be fortified, and additional influential factors should be examined to further enhance iCBT efficacy.

## 12. Recommendations for iCBT for Cancer Patients in Developing Countries

### 12.1 Developing Comprehensive iCBT Frameworks for Clinical Conditions and Fully Automated Software

Currently, iCBTs have been devised for numerous symptoms of breast cancer patients, exhibiting improvement in targeted symptoms as well as other related symptoms, suggesting the potential for addressing entire breast cancer symptom clusters. These patients frequently experience anxiety, depression, sexual dysfunction, fatigue, and insomnia during diagnosis and treatment, often presenting as clusters^135^. Breast cancer symptom clusters primarily encompass fatigue-related symptoms, gastrointestinal symptoms, psychological symptoms, and breast cancer-specific symptoms, with the pain-fatigue-sleep disturbance-depression symptom cluster being the most prevalent and distressing^135^, ^136^. Kwekkeboom et al. discovered that CBT significantly improved not only isolated symptoms such as pain, fatigue, and sleep disturbance, but also the entire symptom cluster^137^. Existing iCBT platforms are predominantly websites, necessitating patients to navigate cumbersome steps like account login and treatment module selection. Voice-controlled software with storage and memory capabilities could facilitate iCBT application. Consequently, future research on iCBT for breast cancer patients should target symptom clusters holistically and develop fully automated iCBT software to attain superior outcomes.

### 12.2 Establishing iCBT Programs Through Multidisciplinary Collaboration Spearheaded by Nurses

A multidisciplinary team (MDT) comprises medical professionals from diverse fields collaborating to develop optimal patient treatment plans^138^. Breast cancer patients require MDT cooperation, with nurses playing a pivotal role^139^. For instance, specialist nurse-led MDTs managing breast cancer patients reduced catheter-related complications, unplanned extubation incidence, and enhanced one-time catheter puncture success rates and patient satisfaction^140^. Mann et al. reported that specialist nurse-provided CBT with relevant training significantly improved breast cancer patient symptoms, with effects lasting beyond six months^141^. Nurse-led iCBT programs may yield superior therapeutic effects and heightened patient satisfaction compared to psychotherapist-exclusive programs, as they more efficiently utilize multidisciplinary medical resources.

## 13. Conclusions

iCBT can effectively ameliorate sexual dysfunction, menopausal symptoms, insomnia, fatigue, and other symptoms in breast cancer patients, but challenges persist: 1) Current iCBT programs typically target specific symptoms; 2) Optimal intervention frequency and duration remain unclear; 3) Control group interventions primarily consist of blank controls or routine care; 4) Psychotherapist workloads are considerable. Therefore, it is advisable to create comprehensive iCBT programs for breast cancer symptom clusters, develop fully automated iCBT software, compare iCBT and CBT therapeutic effects, train breast cancer psychology nurses, and establish nurse-led multidisciplinary teams specializing in breast cancer psychology to collaboratively develop iCBT programs. Future research should acknowledge these limitations and conduct novel investigations on iCBT programs for breast cancer patients.

## Figures and Tables

**Table 1:** Overview of Behavioral Intervention Techniques in Cognitive Behavioral Therapy

Steps	Technique	Description
1	Goal Setting	The therapist aids patients in formulating pragmatic, attainable, and quantifiable objectives, engendering a sense of achievement and self-efficacy, while maintaining focus on constructive endeavors and averting ruminative thinking.
2	Activity Scheduling	The therapist facilitates patients in the systematic organization and planning of daily routines, allotting appropriate time for pleasurable and mastery-oriented activities, thereby fostering positive emotions and feelings of competence.
3	Exposure Therapy	Patients undergo gradual and controlled exposure to anxiety-inducing or distressing situations under the therapist's guidance, enabling confrontation and surmounting of irrational fears or phobias, ultimately attenuating emotional distress.
4	Social Skills Training	The therapist assists patients in the development and refinement of interpersonal communication abilities, assertiveness, and conflict resolution skills, contributing to enhanced social functioning and overall psychological well-being.
5	Problem-Solving Training	Patients are instructed in efficacious problem-solving techniques to adeptly identify, analyze, and address life's challenges, engendering a heightened sense of control and resilience.
6	Mindfulness Techniques	The therapist acquaints patients with mindfulness exercises, such as deep breathing and meditation, cultivating present-moment awareness and non-judgmental acceptance of thoughts and emotions, potentially ameliorating stress and enhancing emotional regulation.
7	Habit Reversal Training	For patients contending with maladaptive habits or compulsive behaviors, therapists employs habit reversal techniques, guiding identification of triggers, implementation of alternative responses, and reinforcement of healthier behavioral patterns.

**Table 2:** Recent Advancements and Novel Developments in Internet-Based Cognitive Behavioral Therapy (iCBT)

Advancements/New Developments	Description
Machine Learning	Machine learning algorithms can personalize iCBT by predicting treatment response and tailoring treatment to individual needs. Machine learning can also be used to automate therapist support and improve treatment outcomes. Machine learning algorithms can analyze user data to predict treatment response, identify risk factors, and personalize therapy based on user characteristics and preferences. Machine learning can also be used to develop chatbots and other digital tools that can provide automated support to users and supplement therapist support^21^, ^22^.
Transdiagnostic iCBT	Transdiagnostic iCBT is a treatment approach that targets multiple disorders with similar underlying mechanisms, such as anxiety and depression. Transdiagnostic iCBT can be delivered through online self-help programs or in conjunction with a therapist and has been found to be effective for reducing symptoms of anxiety and depression. Transdiagnostic iCBT can be more efficient and cost-effective than disorder-specific interventions, as it can address common underlying factors across multiple conditions^23^.
Virtual Reality (VR) iCBT	VR technology is being used to create immersive environments for treating conditions such as social anxiety disorder (SAD) and post-traumatic stress disorder (PTSD). In VR iCBT, users can interact with computer-generated stimuli that simulate real-life situations and experiences, allowing them to practice coping strategies and exposure therapy in a controlled and safe environment^24^.
Metaverse-based iCBT	Metaverse is a term used to describe the convergence of virtual reality and physical reality in a digital space. It is a virtual world in which people can interact through digital selves or avatars^25^. It is possible that in the future, metaverse technology could be used to create immersive environments for delivering iCBT, allowing users to interact with therapy materials and practice coping strategies in a more engaging and interactive way^26^. However, more research is needed to determine the feasibility and effectiveness of metaverse-based iCBT. Overall, while the potential for metaverse-based iCBT is an exciting prospect, it is still in its early stages and more research is needed to determine its feasibility and effectiveness.
Mobile-based iCBT	The use of mobile apps has made iCBT more accessible and convenient for users, allowing them to access therapy on-the-go. Mobile-based iCBT can include interactive exercises, video or audio sessions, and progress tracking to help users manage their mental health from anywhere.^27^.
Guided vs Unguided iCBT	Guided iCBT involves support from a therapist or coach, while unguided iCBT is self-guided without therapist support. Research suggests that guided iCBT may be more effective for depression and anxiety, as therapist support can help users better understand and apply therapy techniques and provide accountability and motivation to complete therapy modules^28^.
Tailored iCBT	Tailored iCBT refers to personalized interventions that are customized based on a user's needs and preferences, including language, culture, and personal goals. Tailored iCBT can improve engagement and treatment outcomes by adapting the content and delivery of therapy to fit the unique needs of each user. Tailored iCBT can be delivered through online self-help programs or in conjunction with a therapist or coach^29^.
Integration with other technologies	iCBT can be integrated with other digital tools, such as wearables, chatbots, and artificial intelligence, to enhance therapy and improve outcomes. Wearable devices can track physiological and behavioral data to provide personalized feedback and insights to users. Chatbots can provide support and guidance to users outside of therapy sessions, while artificial intelligence can personalize therapy by predicting treatment response and tailoring treatment to individual needs^30^.
Stepped Care	Stepped care is a hierarchical model of mental health care that matches the intensity of treatment to the severity of the condition. iCBT can be used as a low-intensity treatment in the initial stages of stepped care and may reduce the need for more intensive treatments later on. In stepped care, users receive treatment that is tailored to their needs and gradually progresses to more intensive levels of care as needed. Stepped care can improve access to mental health care and reduce the burden on healthcare systems by ensuring that users receive the appropriate level of care for their needs^31^.
Blended Care and Blended iCBT	Blended care, a hybrid mental health treatment, merges face-to-face therapy with digital tools like iCBT for effective management of depression, anxiety, and other conditions. This flexible, individualized approach combines digital convenience with personalized in-person sessions. Blended iCBT may involve supplementing therapy with videoconferencing or online programs, or offering support between sessions^32^.
